# Porcelain Teeth

**Published:** 1855-09

**Authors:** H. A. Ackley

**Affiliations:** Cleveland Med. College


*Porcelain Teeth.*—The following note explains itself. It is time that some method was invented that should remove the annoyance caused by the retention of food in the interstices of the ordinary plates, and we have no doubt but a feasible plan would meet with encouragement from our present class of thorough going educated dentists:

Editor of the Buffalo Medical Journal.

Sir:—Allow me to ask you to publish in the Journal the following, which I think worthy the attention and patronage of the profession; it being a great improvement in the mode of making and setting artificial teeth.

They are made to fit individual cases, of one solid piece of porcelain, without the aid of metallic plate of any kind. This mode was invented by Dr. M. Loomis; and a manufactory is now established in Cleveland, Ohio, where full sets, and any considerable parts of sets are being made, and give the wearers universal satisfaction. It is carried on by Drs. Loomis, Wright & Company.

The advantages of this mode are very apparent. It is more cleanly—there being no places for the lodgment of food; more natural—as in this way almost any desired form may be given to the mouth and lips. The absorption consequent on extracting teeth can be entirely remedied; more useful and apparently durable—for no chemical action can, by any possibility, injure them; and much less liable to injury by accident than those set in the usual way. They are cheaper, and we see no reason why this plan will not be universally adopted when understood. *

Cleveland, O., July 30, 1855.

The above I believe to be strictly correct, and have no doubt you will oblige the profession by placing it in your Journal.

H. A. Ackley.

Cleveland Med. College, July 30, 1855.

